# Supplementary material for: De novo pyrimidine biosynthesis inhibition synergizes with BCL-XL targeting in pancreatic cancer
Source: Nat Commun. 2025 Jul 30;16:6987. doi: 10.1038/s41467-025-61242-x (PMC12311037; doi:10.1038/s41467-025-61242-x)
Supplement: Supplementary file 1 — Supplementary Information [file 41467_2025_61242_MOESM1_ESM.pdf]

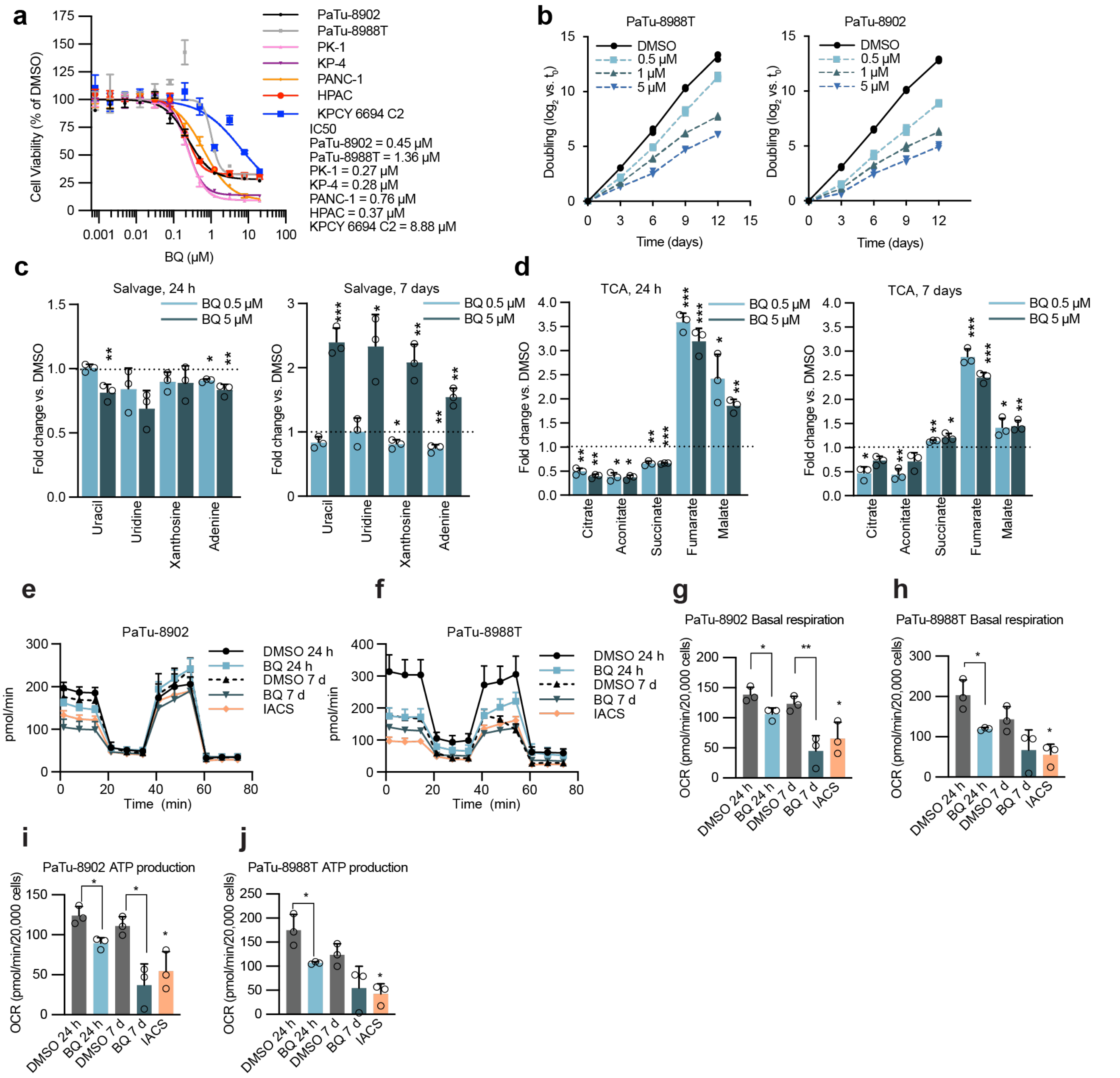

Supplementary Figure 1 Metabolomics and proteomics identify compensatory mechanisms to BQ.

(a) Cell proliferation dose-response curves for PDAC cell lines treated with BQ in DMEM. Error bars represent s.d. of two technical replicates. **(b)** Doubling curves for PaTu-8988T and PaTu-8902 cells treated with BQ (0, 0.5 µM, 1 µM, 5 µM). **(c)** Fold change of metabolites in PaTu-8988T cells in the nucleotide salvage pathway after BQ treatment (24 h: left panel, 7 days: right panel). **(d)** Fold change of metabolites in PaTu-8988T cells in the Tricarboxylic acid (TCA) pathway. Error bars represent s.d. of n = 3 independent plates. Significance determined with t-test for cells treated with BQ vs. vehicle (*$p < 0.05$, **$p < 0.01$, ***$p < 0.001$). **(e-f)** Oxygen Consumption Rate of PaTu-8988T and PaTu-8902 cells treated with BQ for 24 h or 7 days. The complex I inhibitor IACS-010759 was used as a positive control for impaired mitochondrial respiration. **(g-j)** Measurements of basal respiration and ATP production for PaTu-8988T and PaTu-8902 cells. Error bars represent s.d. of average of three independent experiments (each in six technical replicates). Significance was determined with t-test. *$p < 0.05$, **$p < 0.01$.

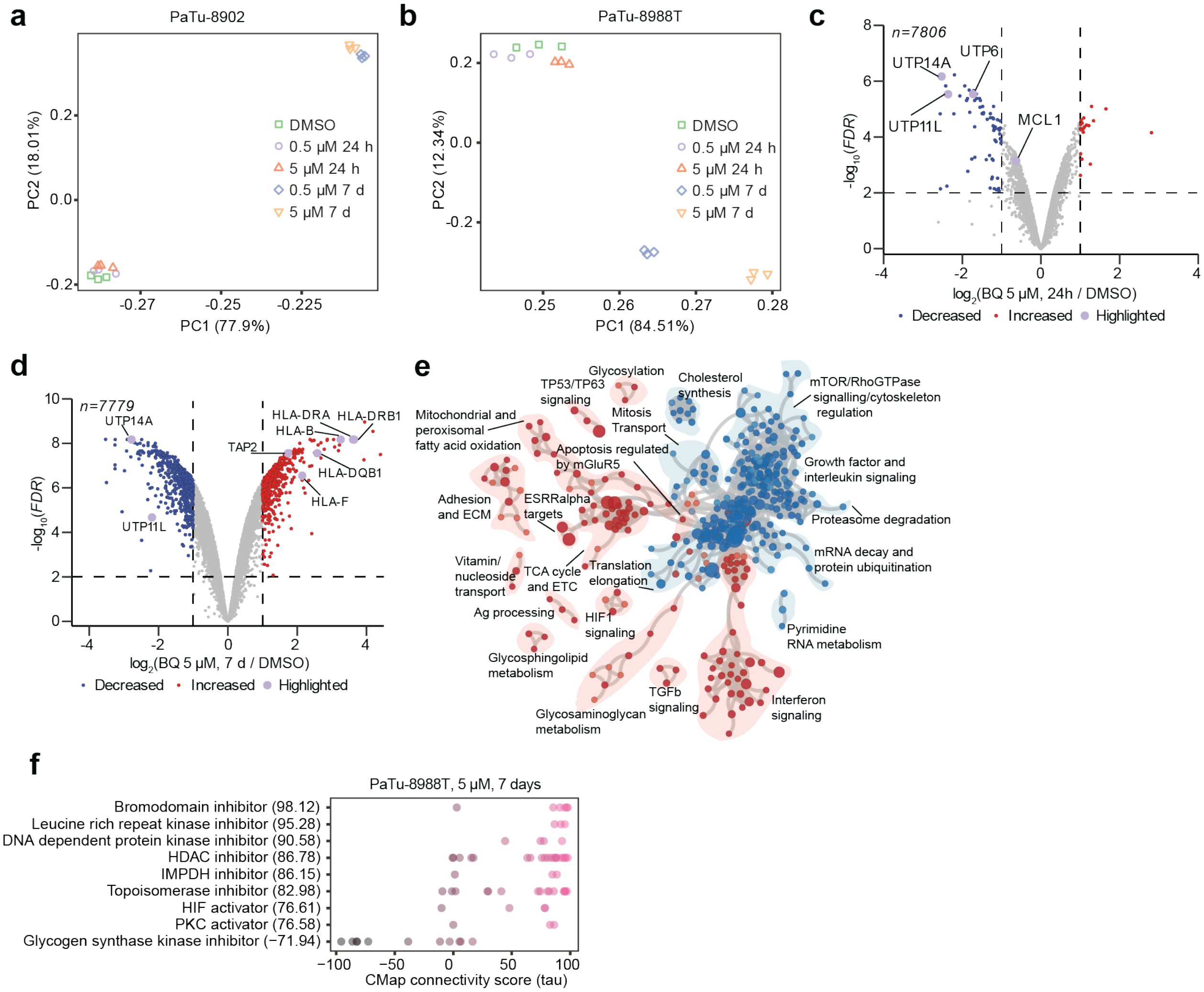

Supplementary Figure 2 Quantitative temporal proteomics of BQ-treated PDAC cells.
(a-b) Principal Component Analysis plots of proteomics data for DMSO or BQ-treated PaTu-8902 **(a)** and PaTu-8988T **(b)** cells show sample clustering. **(c-d)** Volcano plot illustrates significant protein abundance differences in PaTu-8902 cells treated with BQ 5 μM at 24 h **(c)** and 7 days **(d)**. Volcano plots display the $-\log_{10}$ (FDR) versus the $\log_2$ of the relative protein abundance of mean BQ to DMSO-treated samples. Red circles represent significantly upregulated proteins ($\log_2$ fold change≥1, FDR<0.01), whereas blue circles represent significantly downregulated proteins ($\log_2$ fold change≤-1, FDR<0.01). Data from 3 DMSO or 3 BQ-treated independent plates. **(e)** Enrichment map of gene set enrichment analysis (GSEA) of BQ-proteome from PaTu-8902 cells at 7 days (0.5 μM). FDR<0.01, Jaccard coefficient>0.25, node size is related to the number of components identified within a gene set and the width of the line is proportional to the overlap between related gene sets. GSEA terms associated with upregulated (red) and downregulated (blue) proteins are colored accordingly and grouped into nodes with associated terms. **(f)** Connectivity map analysis for PaTu-8988T cells with BQ (5 μM, 7 days). Perturbagen classes with mean connectivity scores >90% or <-90% and FDR≤0.05 are displayed.

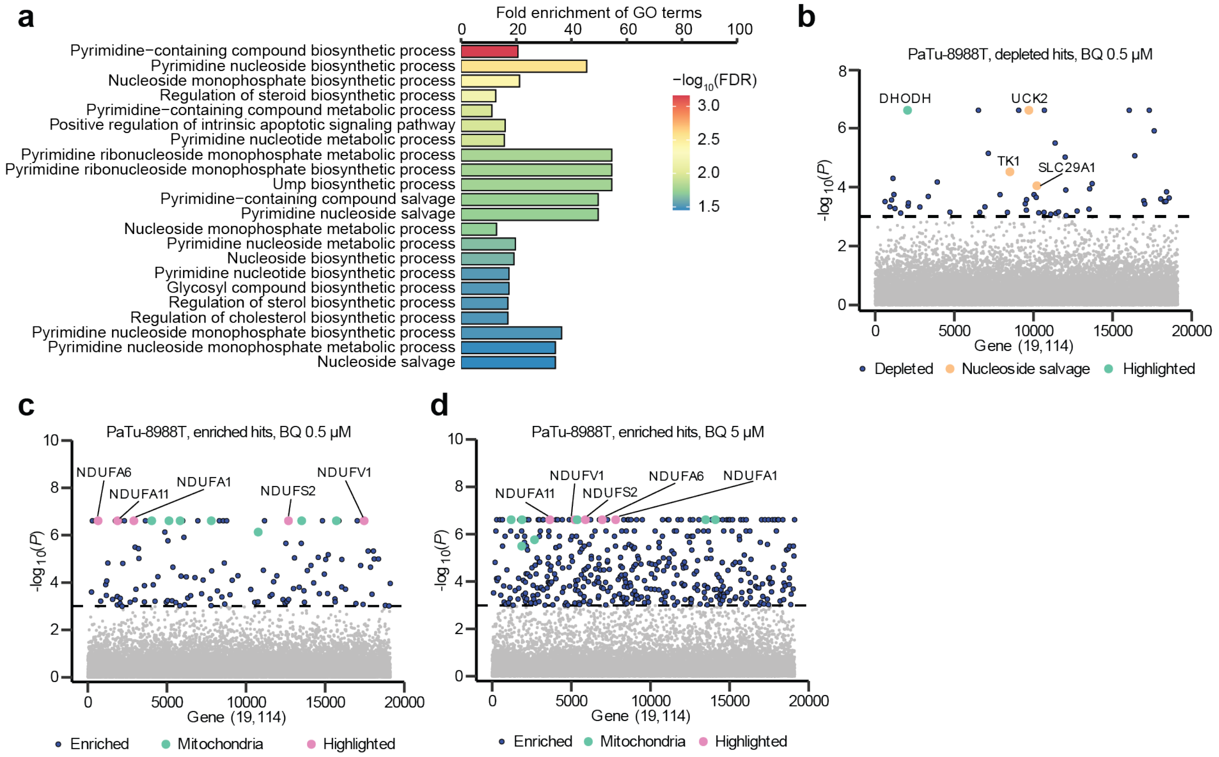

Supplementary Figure 3 BQ-anchored genome-wide CRISPR/Cas9 screen identifies combinatorial drug targets in PDAC cells.

(a) Gene Ontology analysis of the normalized top 100 depleted genes from the in vitro CRISPR/Cas9 screen in PaTu-8988T cells (BQ, 5 μM), fold enrichment is plotted on the x-axis and FDR is denoted by colored bar. **(b-d)** Manhattan plot for in vitro CRISPR/Cas9 screen in PaTu-8988T cells shows **(b)** significant depleted hits at 0.5 μM BQ including nucleoside salvage hits (orange), **(c)** enriched hits at 0.5 μM or **(d)** enriched hits at 5 μM BQ including mitochondrial genes (green, highlighted ones in pink)**.** For all plots, blue dots highlight significant genes ($-\log_{10}(P) \geq 3$) in BQ versus DMSO.

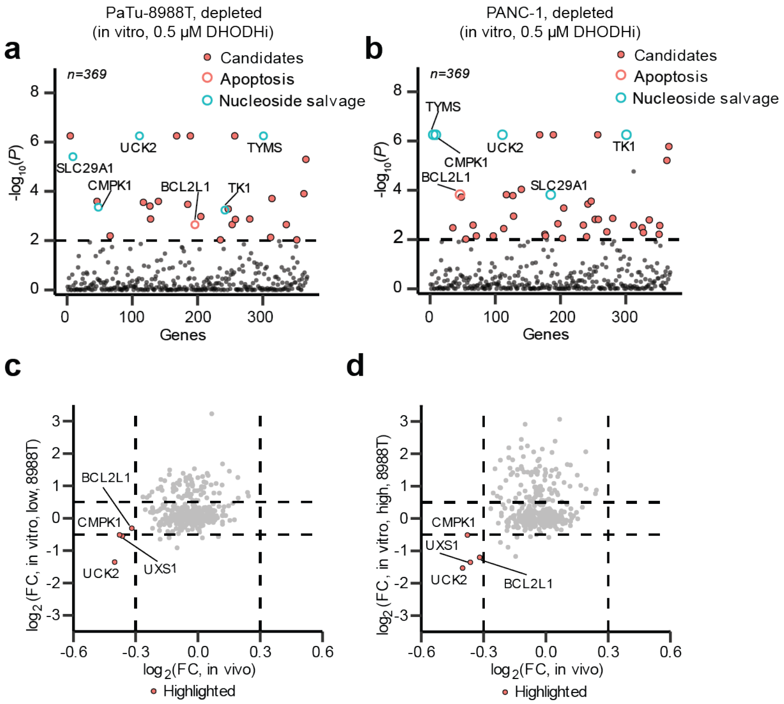

Supplementary Figure 4 BQ-anchored mini-pool loss-of-function CRISPR/Cas9 screen identifies combinatorial drug targets in PDAC cell lines.

(a-b) Manhattan plot for depleted hits in a mini-pool library in vitro CRISPR/Cas9 screen in PaTu-8988T **(a)** and PANC-1 **(b)** cells at 0.5 µM BQ. Orange dots highlight significant genes ($-\log_{10}(P) \geq 2$) in BQ versus DMSO, orange circles highlight genes related to apoptosis and cyan circles highlight genes in the nucleoside salvage pathway. (**c-d**) Correlation plot of depleted or enriched hits in in vitro whole-genome CRISPR/Cas9 screen versus in vivo depleted hits in PaTu-8988T tumors at low or high BQ doses (orange dots highlight significant genes in apoptosis and nucleoside salvage).

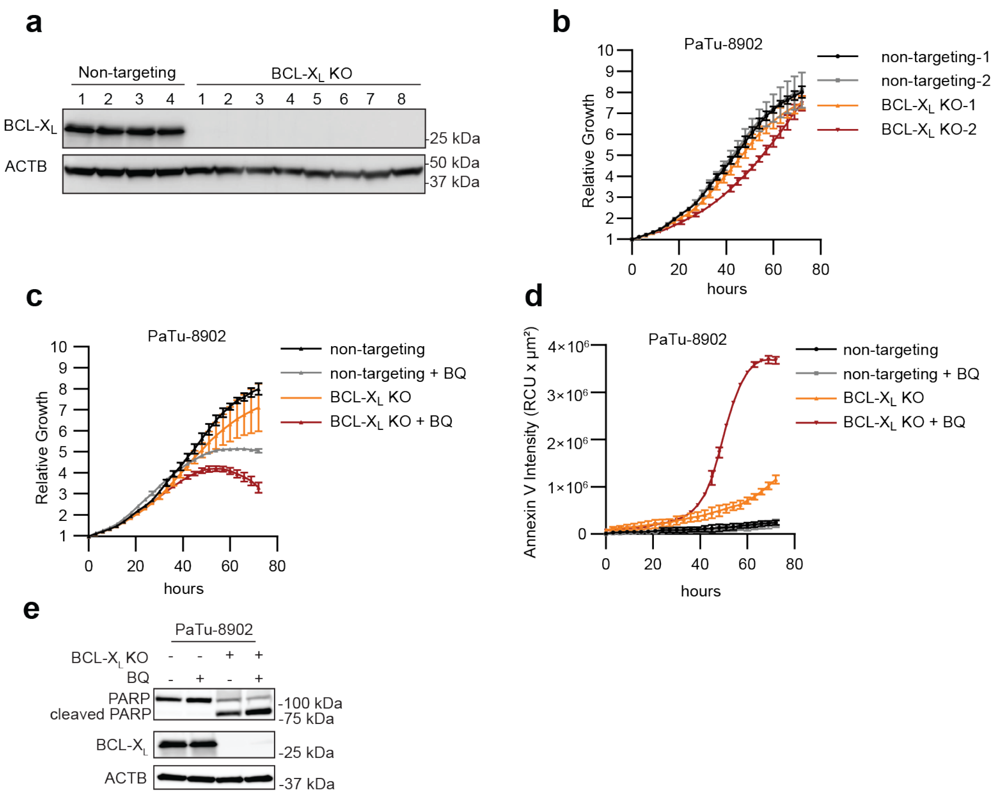

Supplementary Figure 5 BCL-X_L knockout synergizes with BQ to enhance cell death in vitro. **(a)** Immunoblot analysis of BCL-X_L in non-targeting cells and BCL-X_L KO PaTu-8902 cells. The numbers represent individual clones derived from the cell pool following lentivirus infection and subsequent selection. BCL-X_L KO clones 1-4 were derived from the BCL-X_L sgRNA-1 targeting cell pool. BCL-X_L KO clones 5-8 originated from the BCL-X_L sgRNA-2 targeting cell pool. (**b**) Cell proliferation was monitored in real-time using the Incucyte Live-Cell Analysis System and represented as relative growth normalized to day 0 for PaTu-8902 non-targeting cell and BCL-X_L KO over 72 h. Non-targeting-1 and non-targeting-2 refer to clones 1 and 2 in **(a)** labeled as non-targeting. BCL-X_L KO-1 and BCL-X_L KO-2 correspond to clones 1 and 5 from **(a)** labeled as BCL-X_L KO. Clones non-targeting-1 and BCL-X_L KO-1 were used in panels **(c)**, **(d)**, **(e).** Error bars represent s.d. of three technical replicates (representative of two experiments). **(c)** Cell proliferation was monitored in real-time using the Incucyte® Live-Cell Analysis System and represented as relative growth normalized to day 0 for PaTu-8902 non-targeting cell and BCL-X_L KO with or without 5 µM BQ over 72 h. Error bars represent s.d. of three technical replicates (representative of two experiments). **(d)** Real-time accumulation of Annexin V fluorescence in PaTu-8902 non-targeting cells and BCL-X_L KO cells was monitored using the Incucyte system. Cells were labeled with Annexin V Red Dye and treated with 5 µM BQ for 72 hours. Error bars represent s.d. of three technical replicates (representative of two experiments). **(e)** Immunoblot analysis of BCL-X_L and cleaved PARP in non-targeting cells and BCL-X_L KO PaTu-8902 with or without 5 µM BQ.

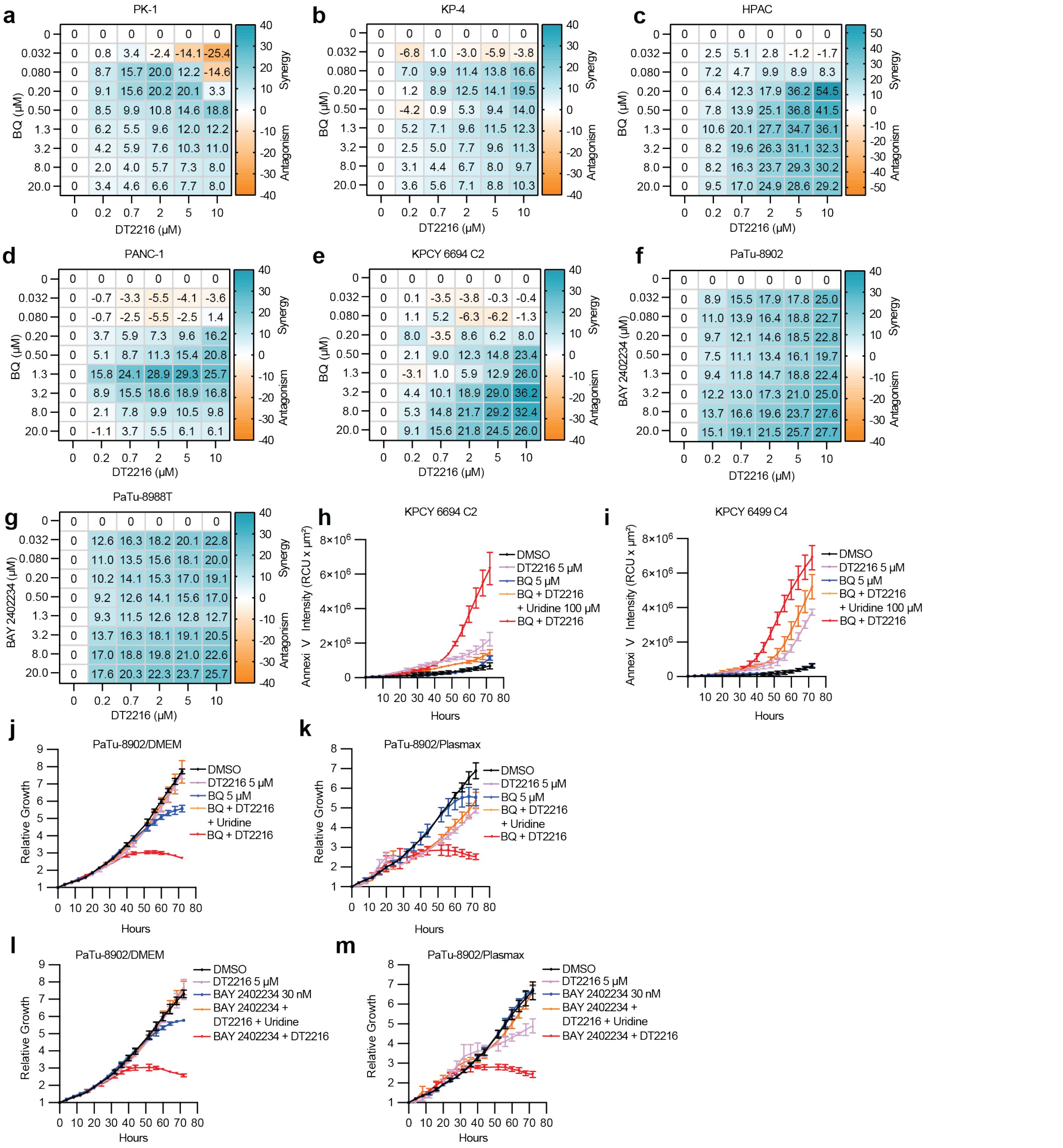

Supplementary Figure 6 BQ and DT2216 combination demonstrates synergy in PDAC cell lines. **(a-g)** Synergy score heatmaps of combination treatment with DT2216 and BQ **(a-e)** or DT2216 and BAY2402234 **(f-g)** in PDAC cells. Synergy score between the two drugs was calculated using the HSA model implemented in SynergyFinder (antagonism: <− 10; additive effect: from − 10 to 10; synergistic effect: >10). Data shown as mean HSA score of three technical replicates. **(h-i)** Real-time accumulation of Annexin V fluorescence in KPCY 6694 C2 and KPCY 6499 C4 cells was monitored using the Incucyte system. Cells were labeled with Annexin V Red Dye and treated with the indicated concentrations of BQ or DT2216 (alone or in combination) or in combination with uridine (100 μM) for 72 hours. Error bars represent s.d. of three technical replicates, representative of two independent experiments. **(j-k)** Cell proliferation was monitored in real-time using the Incucyte® Live-Cell Analysis System and represented as relative growth normalized to day 0 for PaTu-8902 cells in different media (DMEM **j**, Plasmax **k**) treated

with the indicated concentrations of BQ or DT2216 alone or BQ and DT2216 or BQ and DT2216 in combination with uridine (100 μM) for 72 hours. Error bars represent s.d. of three technical replicates (two for the BQ + DT2216 + Uridine condition), representative of two independent experiments. **(l-m)** Cell proliferation was monitored in real-time using the Incucyte® Live-Cell Analysis System and represented as relative growth normalized to day 0 for PaTu-8902 cells in different media (DMEM **l**, Plasmax **m**) treated with the indicated concentrations of BAY 2402234 or DT2216 alone or BAY 2402234 and DT2216 or BAY 2402234 and DT2216 in combination with uridine (100 μM) for 72 hours. Error bars represent s.d. of three technical replicates (two for the BAY 2402234 + DT2216 + Uridine condition), representative of two independent experiments.

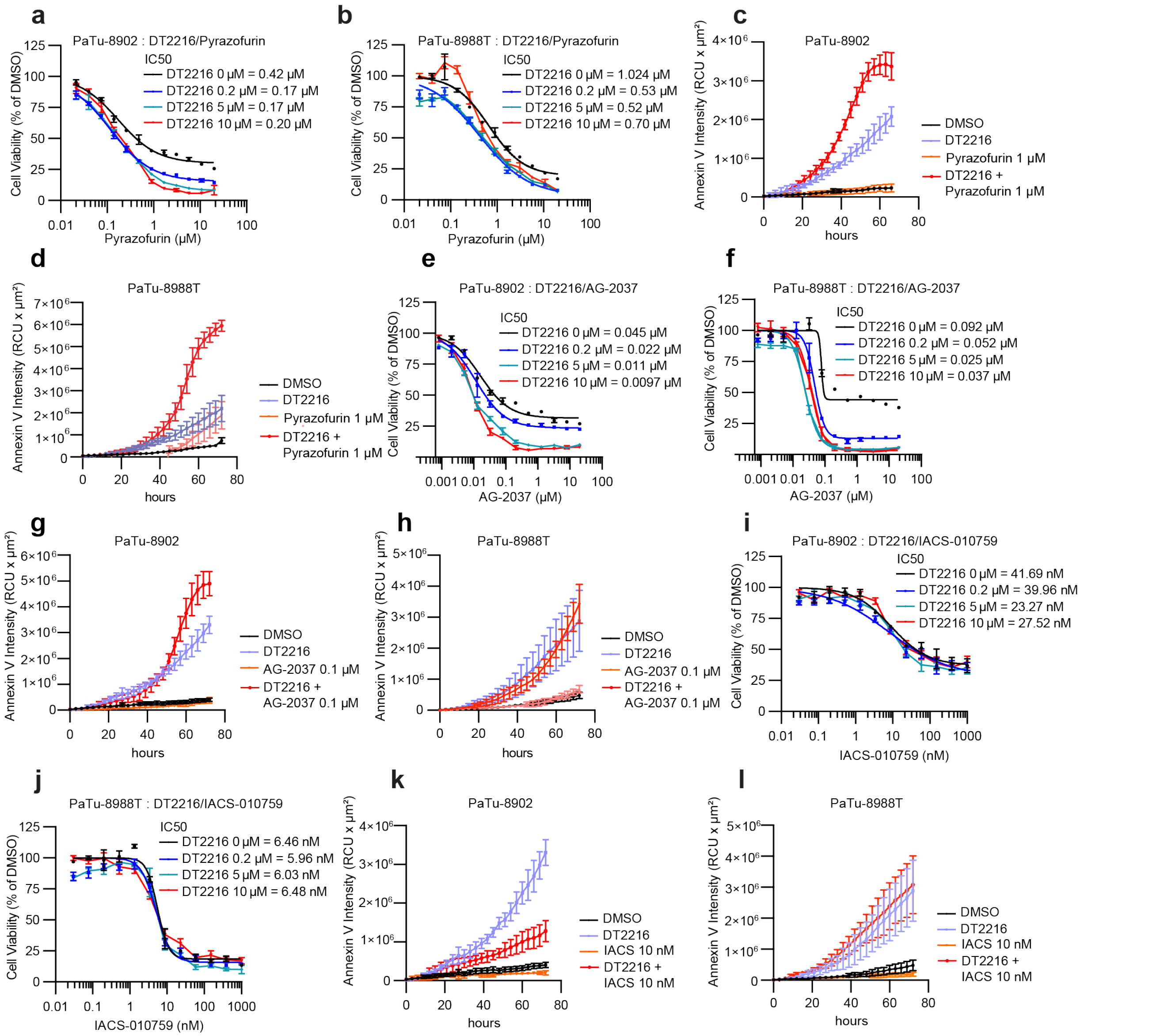

Supplementary Figure 7 Targeting pyrimidine synthesis with UMPS inhibition sensitizes to BCL-X_L targeting.

(a-b) Percentage cell viability of PDAC cells after treatment with increasing concentrations of Pyrazofurin with DT2216 for 5 days. IC50 values are shown for a representative experiment of two independent experiments. Error bars represent s.d. of three technical replicates (two for the DT2216 0 μM condition). **(c-d)** Real-time accumulation of Annexin V fluorescence in PaTu-8902 and PaTu-8988T cells monitored using Incucyte. Cells were labeled with Annexin V Red Dye and treated with DT2216 (1 μM) or Pyrazofurin (1 μM) alone or in combination. Error bars represent s.d. of three technical replicates, representative of two experiments. **(e-f)** Percentage viability of PaTu-8902 and PaTu-8988T cells after treatment with AG-2037 / DT2216 for 5 days, as in (a-b). IC50 values are shown for a representative experiment of two. Error bars represent s.d. of three technical replicates (two for the DT2216 0 μM condition). **(g-h)** Real-time accumulation of Annexin V fluorescence in PaTu-8902 and PaTu-8988T cells was monitored as in (c-d) with DT2216 (2 μM) or AG-2037 (0.1 μM) alone or in combination. Error bars represent s.d. of three technical replicates, representative of two experiments. **(i-j)** Percentage cell viability of PDAC cells after treatment with increasing concentrations of IACS-010759 with DT2216 for 5 days. IC50 values are shown for a representative experiment of two independent experiments. Error bars represent s.d. of three technical replicates (two for the DT2216 0 μM condition). **(k-l)** Real-time accumulation of Annexin V fluorescence in PaTu-8902 and PaTu-8988T cells was monitored as in (c-d) with DT2216 (2 μM) or IACS-010759 (10 nM) alone or in combination for 72 hours. Error bars represent s.d. of three technical replicates (representative of two experiments). DMSO and DT2216 data are shared between (g) and (k), as the combination experiments in PaTu-8902 cells were done side by side with the

same DMSO and DT2216 controls. Similarly, DMSO and DT2216 data are shared between (h) and (l), as the combination experiments in PaTu-8999T cells were done side by side with the same DMSO and DT2216 controls.

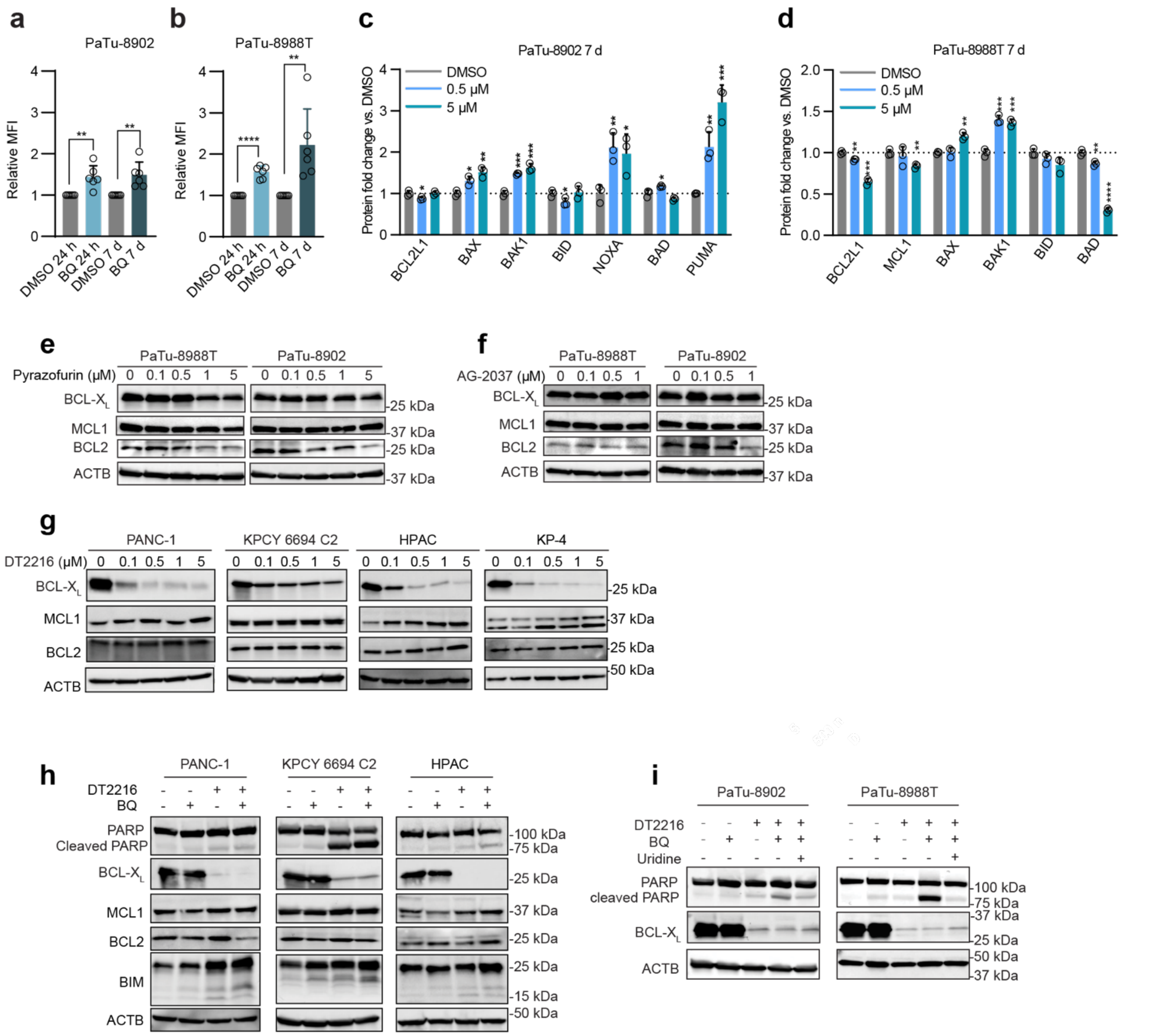

Supplementary Figure 8 Mitochondrial membrane potential and BCL-2 family protein levels after nucleotide synthesis inhibition alone or with DT2216 combination treatment.

(a-b) Mitochondrial membrane potential was measured in PaTu-8902 **(a)** and PaTu-8988T **(b)** after 24 h or 7 days of treatment with Brequinar by flow cytometry. Mean fluorescence Intensity (MFI) of TMRM (Tetramethylrhodamine methyl ester) was calculated by subtracting fluorescence of FCCP-treated cells, normalizing to mitochondrial content measured with MitoTracker Green and normalized to DMSO. Error bars represent s.d. of average of 6 independent experiments. Significance was determined by t-test. ***p* < 0.01, ****p* < 0.001, *****p* < 0.0001. **(c-d)** Proteomics data derived from Fig. 1e and Supplementary Fig. 2d of BCL-2 family protein levels in PaTu-8902 and PaTu-8988T cells treated with 5 μM BQ for 7 days (fold change vs. DMSO). Statistical significance was determined by t-test. **p* < 0.05, ***p* < 0.01, ****p* < 0.001. **(e)** Immunoblot analysis of BCL-2 family proteins in lysates from PaTu-8902 and PaTu-8988T cells treated with indicated concentrations of Pyrazofurin for 24 h. **(f)** Immunoblot analysis of BCL-2 family proteins in lysates from PaTu-8902 and PaTu-8988T cells treated with indicated concentrations of AG-2037 for 24 h. **(g)** Immunoblot analysis of BCL-X_L, MCL1 and BCL2 in lysates from PANC-1, KPCY 6694 C2, HPAC, and KP-4 cells treated with DMSO or DT2216 with indicated doses for 16 hours. **(h)** Immunoblot analysis of BCL-2 family proteins in lysates from indicated cells treated with DMSO or with DT2216 (5 μM) or BQ (5 μM) alone or in combination for 24 hours. **(i)** Immunoblot analysis of BCL-X_L and cleaved PARP in lysates from PaTu-8902 and PaTu-8988T cells treated with DMSO, DT2216 (5 μM) or BQ (μM) alone or in combination with or without uridine (100 μM) for 24 hours.

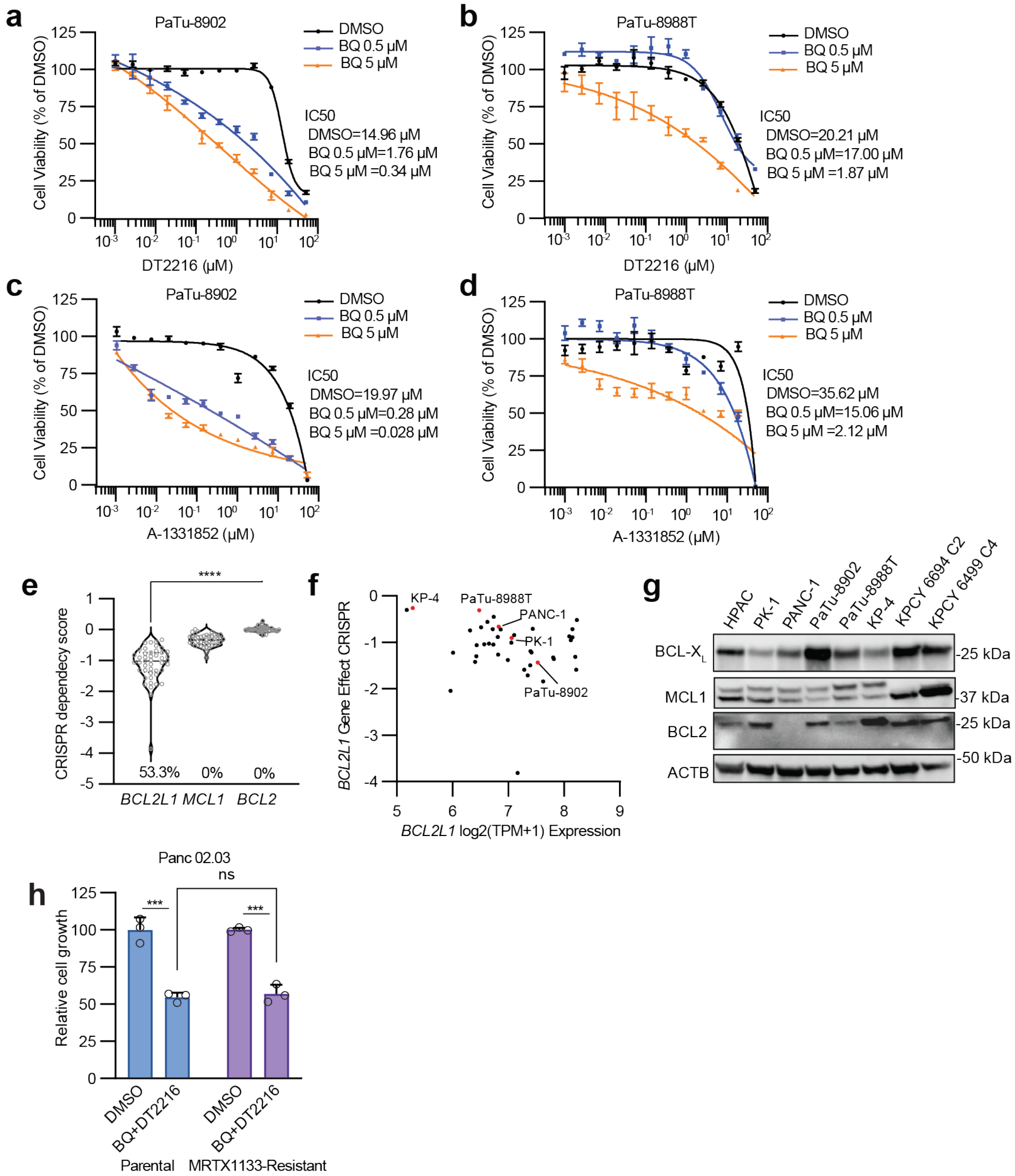

Supplementary Figure 9 BCL-X$_L$ is a dependency in PDAC cell lines and BQ selectively increases sensitivity to BCL-X$_L$ inhibitors in PDAC cell lines.

(a-b) Percentage cell viability of PaTu-8902 and PaTu-8988T cells after treatment with increasing concentrations of DT2216 with DMSO, BQ 0.5 μM or BQ 5 μM for 5 days. IC50 values are shown for a representative experiment out of three independent experiments. Error bars represent s.d. of three technical replicates. **(c-d)** Percentage viability of PaTu-8902 and PaTu-8988T cells treated with increasing concentrations of A-1331852 in combination with DMSO, BQ 0.5 μM or BQ 5 μM for 5 days. IC50 values are shown for a representative experiment out of three independent experiments. **(e)** Plot of CRISPR dependency scores of *BCL2*, *MCL1* and *BCL2L1* in PDAC cell lines in the Cancer Dependency Map (DepMap) (n = 45); dashed line: median value; dotted line: quartile values. Percentage of cell lines scored as dependent (Dependency score < −1) indicated at bottom of graph (****$p < 0.0001$ for all comparisons to *BCL2L1* using one-way ANOVA test)**. (f)** Plot of CRISPR dependency scores and mRNA expression level of *BCL2L1* of PDAC cell lines in the Cancer Dependency Map (n = 43); red dots indicate the cell lines evaluated in this study. **(g)** Immunoblot analysis of BCL-X$_L$, BCL2, and MCL1 in lysates from PDAC cell lines, as indicated. **(h)** Relative growth of Panc 02.03 and Panc 02.03 MRTX1133-resistant cells treated with vehicle (DMSO) or BQ and DT2216 for 3 days. Statistical significance was determined by t-test. ***$p < 0.001$.

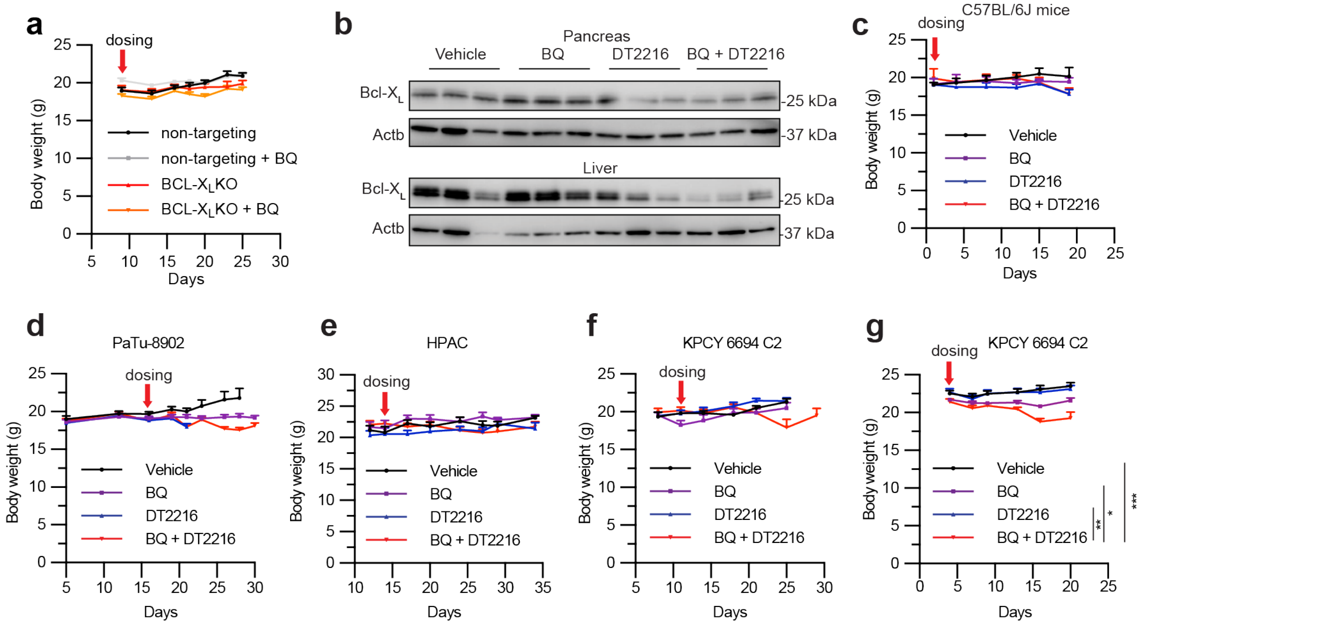

Supplementary Figure 10 Tolerability evaluation of BQ and DT2216 combination therapy in vivo. **(a)** Body weight from (**Fig. 6f**) PaTu-8902 non-targeting and BCL-X_L KO tumors bearing mice after 3 weeks dosing. **(b)** Immunoblot analysis of Bcl-X_L in pancreas and liver from non-tumor bearing C57Bl/6J mice treated with vehicle, DT2216, BQ, or BQ and DT2216 in combination as in **(c). (c)** Body weight changes of non-tumor bearing C57Bl/6J mice treated with vehicle, DT2216, BQ, or BQ and DT2216 for three weeks. DT2216 (15 mg/kg, twice per week) and BQ (10 mg/kg, three times per week) were administered intraperitoneally for three weeks. **(d-g)** Body weight of tumor-bearing models treated as indicated and as presented in **Figure 6**. Mice were treated with vehicle, BQ (10 mg/kg), DT2216 (15 mg/kg), or their combination for 3 weeks. **(d)** PaTu-8902 flank xenograft, **(e)** HPAC flank xenograft, **(f)** KPCY 6694 C2 flank syngeneic allograft, **(g)** KPCY 6694 C2 orthotopic syngeneic allograft. Error bars represent s.e.m. Statistical significance **(g)** was determined by one-way ANOVA. $*p < 0.05$, $**p < 0.01$, $***p < 0.001$.

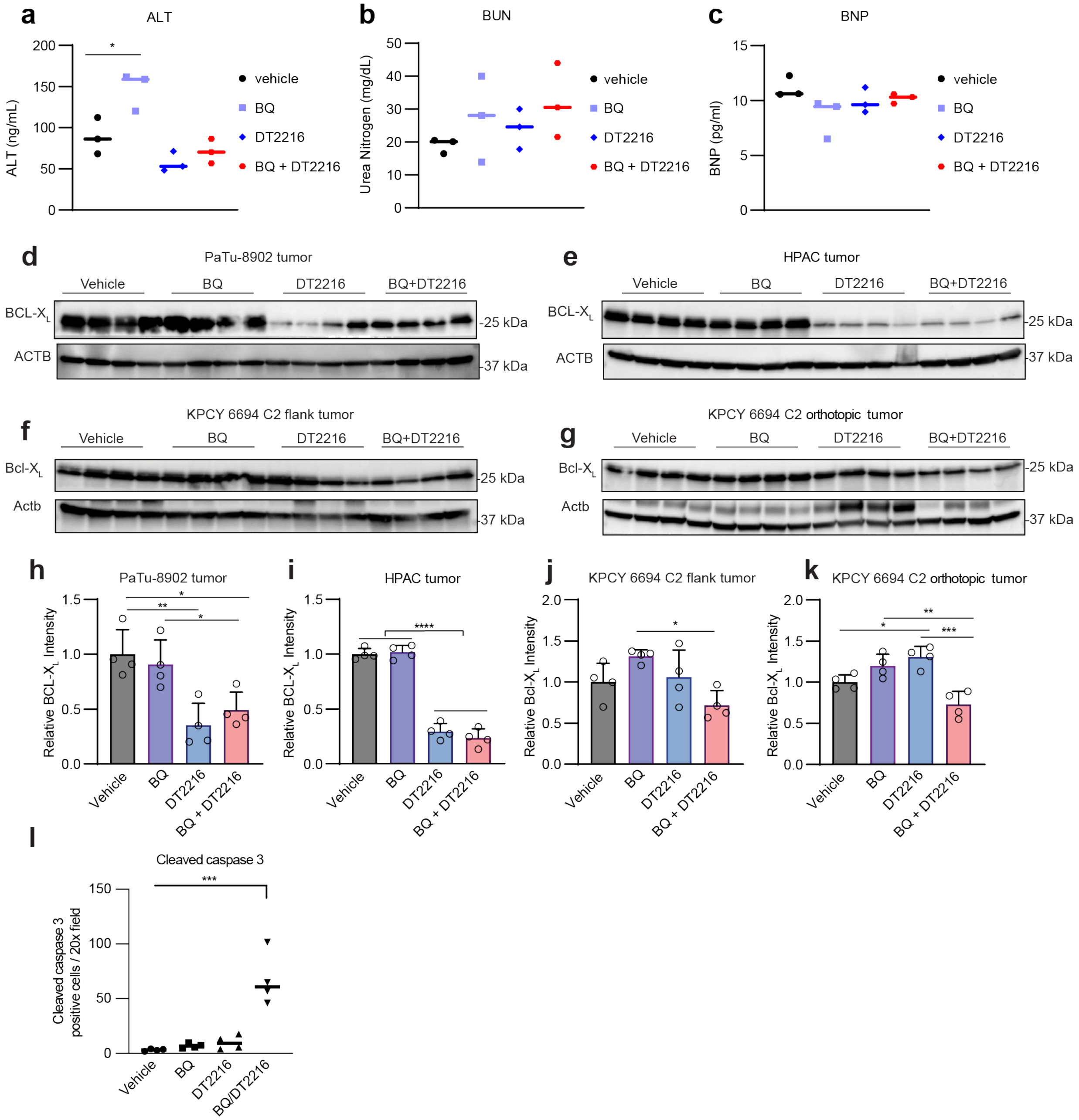

Supplementary Figure 11 Toxicity evaluation of BQ and DT2216 combination therapy in vivo.
(a) Serum levels of alanine aminotransferase (ALT) in tumor-bearing C57BL/6J mice following treatment with Vehicle, BQ (10 mg/kg), DT2216 (15 mg/kg), or their combination. Error bars represent s.d. of 3 mice. **(b)** Serum levels of blood urea nitrogen (BUN) in tumor-bearing C57BL/6J mice following treatment with Vehicle, BQ (10 mg/kg), DT2216 (15 mg/kg), or their combination (normal range in C57/BL6J = 2–71 mg/dL). Error bars represent s.d. of 3 mice. **(c)** Serum levels of brain natriuretic peptide (BNP) in tumor-bearing C57BL/6J mice following treatment with Vehicle, BQ (10 mg/kg), DT2216 (15 mg/kg), or their combination. Error bars represent s.d. of 3 mice. **(d-g)** Immunoblot analysis of BCL-X_L levels in xenograft and syngeneic tumors treated with vehicle, DT2216, BQ, or BQ and DT2216 in combination for 3 weeks. **(h-k)** Quantification of immunoblots in **(d-g)**. **(l)** Number of cleaved caspase 3 positive cells was quantified in 4 fields (20X) from each tumor (PaTu-8902 tumor, n = 4 tumors per group). Each dot represents average of 4 fields from each mouse. Statistical significance was determined by one-way ANOVA. *$p < 0.05$, **$p < 0.01$, ***$p < 0.001$, ****$p < 0.0001$.

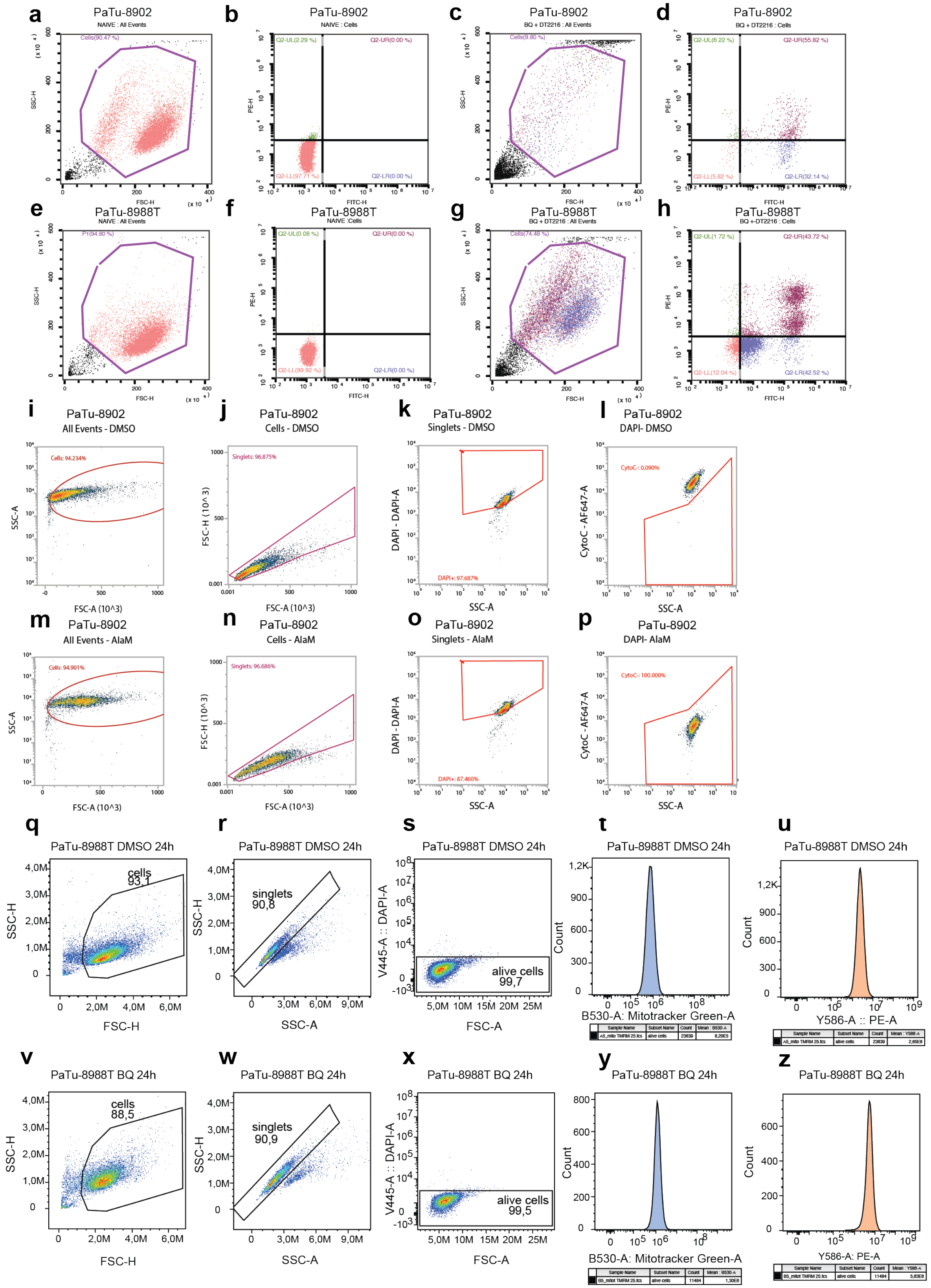

Supplementary Figure 12 Gating scheme of flow cytometry in Fig. 3, Fig. 4 and Supp. Fig. 1. **(a-h)** Representative gating strategy for flow cytometry analysis of apoptotic cell death in PaTu-8902 **(a-d)** and PaTu-8988T cells **(e-h)**, as shown in **Fig. 3c-d**. Naïve, unstained cells were used to define negative control. Cells within the designated quadrants (Annexin V (FITC, x-axis)-positive / PI (PE, y-axis)-negative and Annexin V / PI-positive) were classified as apoptotic. **(i-p)** Gating strategy for flow cytometry analysis of Cytochrome c (cytoC) release shown in **Fig. 4a-b** in PaTu-8902 treated with DMSO **(i-l)** and AlaM **(m-p)**. Apoptosis inducer Alamethicine was positive control and DMSO was negative control. Cytochrome c expression was assessed in the DAPI-positive, singlet-cell population. **(q-z)**

Representative gating strategy for flow cytometry analysis of mitochondrial membrane potential measured by TMRM staining (**Supplementary Fig. 8a-b**) in PaTu-8988T cells treated with DMSO (**q-u**) or Brequinar (**v-z**) for 24h. Mitotracker green (**t, y**) was used for normalization.

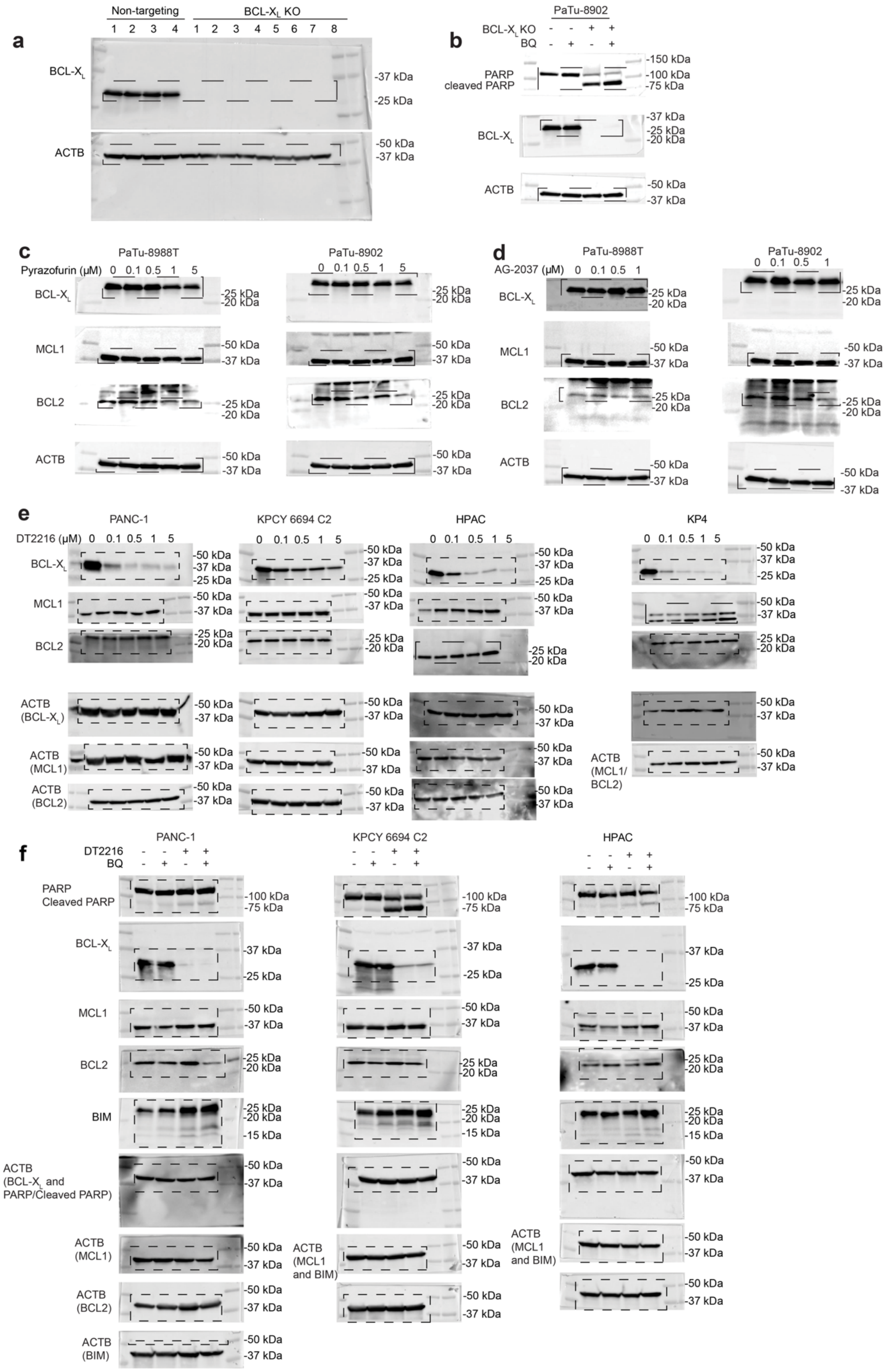

Supplementary Figure 13 Uncropped western blots depicted in Supp. Fig. 5 and Supp. Fig. 8. The segments used in the figures are designated with a dashed frame. **(a)** Supplementary Fig. 5a uncropped blots. **(b)** Supplementary Fig. 5e uncropped blots. **(c)** Supplementary Fig. 8e uncropped blots. **(d)** Supplementary Fig. 8f uncropped blots. **(e)** Supplementary Fig. 8g uncropped blots. **(f)** Supplementary Fig. 8h uncropped blots.

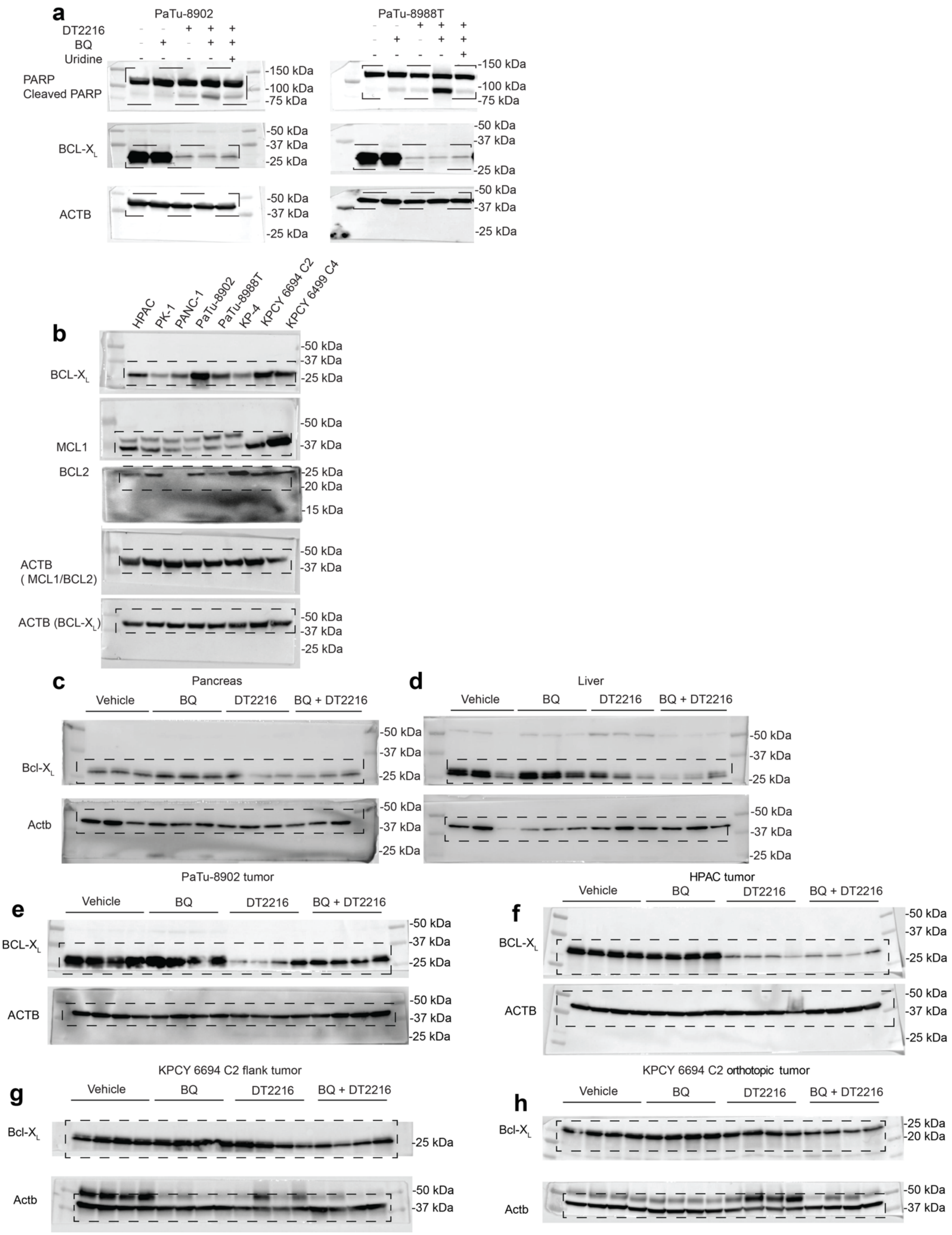

Supplementary Figure 14 Uncropped western blots depicted in Supp. Fig. 8-11. The segments used in the figures are designated with a dashed frame. **(a)** Supplementary Fig. 8i uncropped blots. **(b)** Supplementary Fig. 9g uncropped blots. **(c-d)** Supplementary Fig. 10b uncropped blots. **(e)** Supplementary Fig. 11d uncropped blots. **(f)** Supplementary Fig. 11e uncropped blots. **(g)** Supplementary Fig. 11f uncropped blots. **(h)** Supplementary Fig. 11g uncropped blots.
