# Supplementary material for: De novo pyrimidine biosynthesis inhibition synergizes with BCL-XL targeting in pancreatic cancer
Source: Nat Commun. 2025 Jul 30;16:6987. doi: 10.1038/s41467-025-61242-x (PMC12311037; doi:10.1038/s41467-025-61242-x)
Supplement: Supplementary file 2 — Description of Additional Supplementary Files [file 41467_2025_61242_MOESM2_ESM.pdf]

Description of Additional Supplementary Files

Supplementary Data 1: LC-MS/MS peak area values of steady state metabolomics from BQ-treated PaTu-8988T (24h and 7 days) as represented in Fig. 1, and Supplementary Fig. 1.

Supplementary Data 2: Proteome changes of PA-TU-8988t and PA-TU-8902 cells after BQ treatment. limma package applies linear modeling with empirical Bayes moderation to improve variance estimates across proteins, and adjusted p-values is calculated via Benjamini-Hochberg method.

Supplementary Data 3: GSEA analysis of proteome changes in Table 2. GSEA performs permutation testing, then computes FDR q-values to correct for multiple testing across many gene sets

Supplementary Data 4: Summary of sgRNA coverage across CRISPR screens in this study.

Supplementary Data 5: MAGeCK analysis results of BQ-anchored genome-wide CRISPR screen. MAGeCK uses a negative binomial model to analyze sgRNA count data and applies Robust Rank Aggregation (RRA) to score gene-level effects. Adjusted p-value is calculated using Benjamini-Hochberg method.

Supplementary Data 6: Raw read counts of CRISPR screens in this study.

Supplementary Data 7: MAGeCK analysis results of BQ-anchored in vitro and in vivo mini-pool CRISPR screen.

Supplementary Data 8: Organoid information

Supplementary Data 9: Blood hematology analysis in NOD.Cg-Prkdcscid Il2rgtm1Sug/JicTac mice treated with Vehicle and BQ for 3 weeks

Supplementary Data 10: Blood hematology analysis in C57BL/6J treated with Vehicle, BQ, DT2216 and BQ combination with DT2216 for 3 weeks

Supplementary Data 11: Blood hematology analysis in NOD.Cg-Prkdcscid Il2rgtm1Sug/JicTac mice treated with Vehicle, BQ, DT2216 and BQ combination with DT2216 for 3 weeks

Supplementary Data 12: sgRNA sequences and primers used for qRT-PCR
