# Supplementary material for: De novo pyrimidine biosynthesis inhibition synergizes with BCL-XL targeting in pancreatic cancer
Source: Nat Commun. 2025 Jul 30;16:6987. doi: 10.1038/s41467-025-61242-x (PMC12311037; doi:10.1038/s41467-025-61242-x)
Supplement: Supplementary file 15 — Reporting Summary [file 41467_2025_61242_MOESM15_ESM.pdf]

nature portfolio

Corresponding author(s): Joseph D. Mancias and Naiara Santana-Codina

Last updated by author(s): Jun 2, 2025

Reporting Summary

Nature Portfolio wishes to improve the reproducibility of the work that we publish. This form provides structure for consistency and transparency in reporting. For further information on Nature Portfolio policies, see our Editorial Policies and the Editorial Policy Checklist.

Statistics

For all statistical analyses, confirm that the following items are present in the figure legend, table legend, main text, or Methods section.

n/a	Confirmed	
☐	☒	The exact sample size (n) for each experimental group/condition, given as a discrete number and unit of measurement
☐	☒	A statement on whether measurements were taken from distinct samples or whether the same sample was measured repeatedly
☐	☒	The statistical test(s) used AND whether they are one- or two-sided<br>*Only common tests should be described solely by name; describe more complex techniques in the Methods section.*
☒	☐	A description of all covariates tested
☒	☐	A description of any assumptions or corrections, such as tests of normality and adjustment for multiple comparisons
☐	☒	A full description of the statistical parameters including central tendency (e.g. means) or other basic estimates (e.g. regression coefficient) AND variation (e.g. standard deviation) or associated estimates of uncertainty (e.g. confidence intervals)
☐	☒	For null hypothesis testing, the test statistic (e.g. F, t, r) with confidence intervals, effect sizes, degrees of freedom and P value noted<br>*Give P values as exact values whenever suitable.*
☐	☒	For Bayesian analysis, information on the choice of priors and Markov chain Monte Carlo settings
☒	☐	For hierarchical and complex designs, identification of the appropriate level for tests and full reporting of outcomes
☒	☐	Estimates of effect sizes (e.g. Cohen's d, Pearson's r), indicating how they were calculated

Our web collection on statistics for biologists contains articles on many of the points above.

Software and code

Policy information about availability of computer code

Data collection	ChemiDocTM MP Imager (Bio-Rad) for western blot data collection<br>Beckman Coulter (CytoFLEX S), Attune NxT flow cytometer and NovoCyte Quanteon analyzer (Agilent Technologies, Santa Clara, CA, USA) - flow cytometry data collection<br>Seahorse: Wave 2.6.1 (for Seahorse XFe96 Flux Analyzer) and Cell Imaging Software (for Cytation1) by Agilent Technologies (1.1.0.17) - for respiration measurements<br>Incucyte® Live-Cell Analysis System - for growth and annexin real-time measurements<br>Orbitrap Lumos mass spectrometer in line with a Proxeon NanoLC-1200 UHPLC system for Proteomics<br>5500 QTRAP hybrid dual quadrupole ion trap mass spectrometer (AB/SCIEX) and a Prominence HPLC (Shimadzu) with an autosampler outfitted with an Amide XBridge column (Waters; 3.5 µm particle size, 4.6 mm diameter (i.d.) x 100 mm length; Waters cat. No. 186004868) for metabolomics.Software for peak integration: MultiQuant v2.0 (AB/SCIEX)<br>QuantStudio 7 Flex-for qPCR<br>Amersham Imager 600 for Western Blot scanning<br>CLARIOstar from BMGLABTECH for CellTiter-Glo based viability assay
Data analysis	R vsoftware (4.0.3) and LIMMA package (3.40.2) - for proteomics analysis<br>Broad GSEA software (3.0) - for proteomics data analysis<br>Cytoscape (3.7.2) with EnrichmentMap (3.2.1) - for proteomics data analysis<br>Connectivity Map (CMap) 2.0.<br>Adobe Illustrator (v24.1.2)<br>Image J 1.52a; Java 1.8.0

GraphPad Prism 10.4.0
FlowJo™ v10.10 Software (BD Life Sciences).
Beckman Coulter (CytoFLEX S) and Attune NxT flow cytometer analysis system

For manuscripts utilizing custom algorithms or software that are central to the research but not yet described in published literature, software must be made available to editors and reviewers. We strongly encourage code deposition in a community repository (e.g. GitHub). See the Nature Portfolio guidelines for submitting code & software for further information.

Data

Policy information about availability of data

All manuscripts must include a data availability statement. This statement should provide the following information, where applicable:

- Accession codes, unique identifiers, or web links for publicly available datasets
- A description of any restrictions on data availability
- For clinical datasets or third party data, please ensure that the statement adheres to our policy

Data availability:
- Proteomic data are available in Supplementary Data 2 and have been deposited to the ProteomeXchange consortium via the PRIDE partner repository. Data are available with the identifier PXD059471
- CRISPR screen data are available on Supplementary Data 4-6 and at the European Nucleotide Archive (ENA) under the following accession number PRJEB90251
- Metabolomics data are available in Supplementary Data 1 and have been deposited to the MassIVE repository with the dataset identifier MSV000097577.

Research involving human participants, their data, or biological material

Policy information about studies with human participants or human data. See also policy information about sex, gender (identity/presentation), and sexual orientation and race, ethnicity and racism.

Reporting on sex and gender	*Use the terms sex (biological attribute) and gender (shaped by social and cultural circumstances) carefully in order to avoid confusing both terms. Indicate if findings apply to only one sex or gender; describe whether sex and gender were considered in study design; whether sex and/or gender was determined based on self-reporting or assigned and methods used.*<br>*Provide in the source data disaggregated sex and gender data, where this information has been collected, and if consent has been obtained for sharing of individual-level data; provide overall numbers in this Reporting Summary. Please state if this information has not been collected.*<br>*Report sex- and gender-based analyses where performed, justify reasons for lack of sex- and gender-based analysis.*
Reporting on race, ethnicity, or other socially relevant groupings	No report on race, ethnicity or other socially relevant groupings was used in this study.
Population characteristics	No population characteristics were used in this study.
Recruitment	The organoids were derived from tissue specimens from patients with metastatic or localized PDAC who underwent resection or biopsy between November 2015 and July 2019 at DF/BWCC. Investigators obtained written, informed consent from patients at least 18 years old with pancreatic cancer for Dana-Farber / Harvard Cancer Center IRB-approved protocols 11–104, 17–000, 03–189, and/or 14–408 for tissue collection, molecular analysis, and organoid generation. Relevant population characteristics of the human research participants wer previously defined (Raghavan, Cell, 2021).
Ethics oversight	The study design was approved by the IRB at Dana-Farber (protocols 11–104, 17–000, 03–189, and/or 14–408). The study was conducted in accordance with the U.S. Common Rule.

Note that full information on the approval of the study protocol must also be provided in the manuscript.

Field-specific reporting

Please select the one below that is the best fit for your research. If you are not sure, read the appropriate sections before making your selection.

[x] Life sciences [ ] Behavioural & social sciences [ ] Ecological, evolutionary & environmental sciences

For a reference copy of the document with all sections, see nature.com/documents/nr-reporting-summary-flat.pdf

Life sciences study design

All studies must disclose on these points even when the disclosure is negative.

Sample size	As described previously (PMID: 28671190, PMID: 30470748) no sample size calculation was done either for in vitro or in vivo studies. For in vivo studies, sample sizes were determined based on our preliminary experiments. In our experience, n = 5-10 mice per group is sufficient to detect meaningful biological differences with good reproducibility. Sample sizes were not predetermined based on statistical methods, but were chosen based on standards of the field which provided sufficient statistics to measure size of effect.

Data exclusions	No samples were excluded from analysis.
Replication	All results were tested and confirmed with at least two-three independent experiments.
Randomization	No method of randomization was applied.
Blinding	Blinding was not performed in mouse experiments. because the investigator needed to know the treatment groups in order to perform the study. Tumour weights (an objective measurement) were carried out at the study endpoints after mice were euthanized and tumours were harvested. In vitro studies were not blinded. Experimental and surrogate counting wells had to be assigned and treated similarly.

Reporting for specific materials, systems and methods

We require information from authors about some types of materials, experimental systems and methods used in many studies. Here, indicate whether each material, system or method listed is relevant to your study. If you are not sure if a list item applies to your research, read the appropriate section before selecting a response.

Materials & experimental systems

n/a	Involved in the study
☐	☒ Antibodies
☐	☒ Eukaryotic cell lines
☒	☐ Palaeontology and archaeology
☐	☒ Animals and other organisms
☒	☐ Clinical data
☒	☐ Dual use research of concern
☒	☐ Plants

Methods

n/a	Involved in the study
☒	☐ ChIP-seq
☐	☒ Flow cytometry
☒	☐ MRI-based neuroimaging

Antibodies

Antibodies used

The following antibodies were used for western blotting:
BCL-2 (D17C4) (CST, Cat# 3498, RRID:AB_1903907, 1:500)
BCL-XL (54H6) (CST, #2764, RRID:AB_222800, 1:1000)
MCL-1 (D35A5) (CST, #5453S, RRID:AB_10694494, 1:1000)
PARP (CST, 9542S, RRID:AB_2160739, 1:1000)
PUMA (E2P7G) (CST, #98672S, RRID:AB_3096180, 1:1000)
BIM (C34C5) (CTS, #2933, RRID:AB_1030947, 1:1000)
cleaved caspase 3 (Asp175) (CST, #9661, RRID:AB_2341188, 1:1000)
Phospho-NF-κB p65 (Ser536)(93H1) (CST, #3033S, RRID:AB_331284, 1:1000)
NF-κB p65 (D14E12) (CST, #8242S, RRID:AB_10859369, 1:1000)
Phospho-NF-κB p105 (Ser932) (18E6) (CST, #4806S, RRID:AB_2282911, 1:1000)
NF-κB1 p105 Antibody (CST, #4717S, RRID:AB_2282895, 1:1000)
ACTB (Sigma, A5441, RRID:AB_476744, 1:5,000)

Secondary antibodies were as follows:
Anti-rabbit IgG (H1L) HRP conjugate (Thermo, 31460, RRID:AB_228341, 1:3,000)
Anti-mouse IgG (H1L) HRP conjugate (Promega, W4021, RRID:AB_43083, 1:7,000)

The following was used for IHC:
cleaved caspase 3 (Asp175) (CST, #9661, RRID:AB_2341188, 1:400)

Validation

Antibody specificity for BCL-XL was confirmed using BCL2L1 knockouts as displayed in Supplementary Fig. 5.
Antibody specificity for BCL-2 Antibody was validated by the manufacturing company using extracts from a 293T cell line transfected with mouse-Bcl2. https://www.cellsignal.com/products/primary-antibodies/bcl-2-d17c4-rabbit-mab/3498
Antibody specificity for MCL-1 Antibody was validated by the manufacturing company using extracts from a 293T cell line transfected with human / mouse Mcl-1 constructs. https://www.cellsignal.com/products/primary-antibodies/mcl-1-d35a5-rabbit-mab/5453
Antibody specificity for PARP Antibody was validated by the manufacturing company using extracts from a NIH/3T3 cell line treated with staurosporine and from Jurkat cells treated with etoposide. https://www.cellsignal.com/products/primary-antibodies/parp-antibody/9542
Antibody specificity for PUMA Antibody was validated by the manufacturing company using extracts from HCT116 cells treated with doxorubicin and by Yu et al, PNAS, 2003 using PUMA KO cell lines. https://www.cellsignal.com/products/primary-antibodies/puma-e2p7g-rabbit-mab/98672
Antibody specificity for BIM Antibody was validated by the manufacturing company using extracts from HeLa cells transfected with an siRNA against BIM. https://www.cellsignal.com/products/primary-antibodies/bim-c34c5-rabbit-mab/2933
Antibody specificity for cleaved caspase 3 Antibody was validated by the manufacturing company using extracts from HeLa, NIH/3T3 and C6 cells cells treated with staurosporine. https://www.cellsignal.com/products/primary-antibodies/cleaved-caspase-3-asp175-

antibody/9661?qs=keyword_redirect&qt#9661
Antibody specificity for phospho-NF-κB p65 antibody was validated by the manufacturing company using extracts from HeLa, NIH/3T3 cells treated with TNF-alpha. https://www.cellsignal.com/products/primary-antibodies/phospho-nf-kb-p65-ser536-93h1-rabbit-mab/3033
Antibody specificity for NF-κB p65 antibody was validated by the manufacturing company by confocal immunofluorescent staining showing nuclear localization of the protein after hTNF-alpha stimulation of HT-1080 cells. https://www.cellsignal.com/products/primary-antibodies/nf-kb-p65-d14e12-xp-rabbit-mab/8242
Antibody specificity for Phospho-NF-κB p105 (Ser932) antibody was validated by the manufacturing company using extracts from Vero cells treated with TNF-alpha. https://www.cellsignal.com/products/primary-antibodies/phospho-nf-kb-p105-ser932-18e6-rabbit-mab/4806
Antibody specificity for NF-κB p105 (Ser932) antibody was validated by Zhao et al, Cell, 2018 using adipose tissue from mice stimulated with TNF-alpha.
Antibody specificity for Actin B was validated by manufacturing company: https://www.sigmaaldrich.com/deepweb/assets/sigmaaldrich/product/documents/296/386/a5441dat.pdf?srsltid=AfmBOooee-41qSezWiLMVXfpnlCYCL8OmPBaQKIMws0zGOvwZgxwwsWSv
Anti-rabbit IgG (H+L) HRP conjugate: https://www.thermofisher.com/order/genome-database/dataSheetPdf?producttype=antibody&productsubtype=antibody_secondary&productId=31460&version=Local
anti-mouse IgG (H1L) HRP conjugate was validated by manufacturing company using serial dilutions of lysate: https://www.promega.com/products/protein-detection/primary-and-secondary-antibodies/anti_mouse-igg-h-and-l-hrp-conjugate/?catNum=W4021&cs=y

Eukaryotic cell lines

Policy information about cell lines and Sex and Gender in Research

Cell line source(s)

PaTu-8988T and PaTu-8902 cells were obtained from DSMZ (ACC 162 and ACC 179), PANC-1, HPAC, Panc 02.03 and HEK-293T cells were from ATCC (CRL-1469, CRL-2119, CRL-2553, CRL-3216) and PK-1 and KP-4 were from RIKEN Cell Bank (RBC1972, RBC1005). The murine KPCY 6499 C4 and KPCY6694 C2 cell lines were from Kerafast EUP015-FP and EUP006-FP, respectively.

Sex of the cell lines used:
PaTu-8988T - female
PaTu-8902 - female
PANC-1 - male
HPAC - female
Panc 02.03 - female
HEK-293T - female
PK-1 - male
KP-4 - male
KPCY-6499 C4 - female
KPCY 6694 C2 - female

All organoid used for this study were from female patients:
PANFR0069_T1 – Female
PANFR0165_T3 – Female
PANFR0233_T1 – Female
PANFR0575_T1 – Female

Authentication

Cell lines were maintained in a centralized cell bank and authenticated by assessment of cell morphology as well as short tandem repeat fingerprinting.

Mycoplasma contamination

All the cell lines and organoids used were routinely tested for Mycoplasma contamination using PCR and were negative for Mycoplasma

Commonly misidentified lines
(See ICLAC register)

No commonly misidentified lines were used.

Animals and other research organisms

Policy information about studies involving animals; ARRIVE guidelines recommended for reporting animal research, and Sex and Gender in Research

Laboratory animals

Mus Musculus, NOD.Cg-Prkdcscid Il2rgtm1Sug/JicTac, age 7 and 9 weeks, females (Taconic)
NOD.Cg-Prkdcscid Il2rgtm1Wjl/SzJ mice, 8 weeks, females (Jackson)
C57BL/6J mice, 8 weeks and 10 weeks, females (Jackson)

For all in vivo experiments, mice were housed in pathogen-free animal facilities at Dana-Farber Cancer Institute on standard 12h dark / 12h light cycle under controlled environmental conditions (temperature: 20+/−2°C; humidity: 35+/−10%). Prolab Isopro RMH 3000 diet (Labdiet 5P75 and 5P76) and water were provided ad libitum.

Wild animals

No wild animals were used in this study

Reporting on sex	Mouse and human pancreatic cancer cell lines were derived from both male and females. All the mice for this study were female and sex was not a specified variable for analysis .
Field-collected samples	No field-collected data samples were used in this study
Ethics oversight	All animal experiments were performed in accordance with a Dana-Farber Cancer Institute Institutional Animal Care and Use Committee–approved protocol (10-055).

Note that full information on the approval of the study protocol must also be provided in the manuscript.

Plants

Seed stocks	*Report on the source of all seed stocks or other plant material used. If applicable, state the seed stock centre and catalogue number. If plant specimens were collected from the field, describe the collection location, date and sampling procedures.*
Novel plant genotypes	*Describe the methods by which all novel plant genotypes were produced. This includes those generated by transgenic approaches, gene editing, chemical/radiation-based mutagenesis and hybridization. For transgenic lines, describe the transformation method, the number of independent lines analyzed and the generation upon which experiments were performed. For gene-edited lines, describe the editor used, the endogenous sequence targeted for editing, the targeting guide RNA sequence (if applicable) and how the editor was applied.*
Authentication	*Describe any authentication procedures for each seed stock used or novel genotype generated. Describe any experiments used to assess the effect of a mutation and, where applicable, how potential secondary effects (e.g. second site T-DNA insertions, mosiacism, off-target gene editing) were examined.*

Flow Cytometry

Plots

Confirm that:

- [x] The axis labels state the marker and fluorochrome used (e.g. CD4-FITC).
- [x] The axis scales are clearly visible. Include numbers along axes only for bottom left plot of group (a 'group' is an analysis of identical markers).
- [ ] All plots are contour plots with outliers or pseudocolor plots.
- [ ] A numerical value for number of cells or percentage (with statistics) is provided.

Methodology

Sample preparation	For cell death measurements by PI/Annexin, Cells were plated at 50,000 cells/well (24-well plate). Cells were treated the next day with the indicated compounds for 72 h. Adherent and non-adherent cells in the medium were collected and stained with Annexin-FITC and propidium iodide for 15 min (BD biosciences 556547) as per the manufacturer protocol. Cells were placed on ice and analyzed using a Beckman Coulter Cytoflex.<br><br>For TMRM measurements, human PDAC cell lines treated with brequinar were trypsinized and stained with 25 nM Image-iT TMRM Reagents (Thermo Scientific, #I34361) and 20 µM MitoTracker™ Green (Thermo Scientific, Cat. #M46750) in DMEM supplemented with 1% FBS at 37°C for 30 minutes. FCCP at 1µM was added 10 minutes prior to analysis as positive control.<br><br>For BH3 profiling, cultured cells were trypsinized, centrifuged at 500xg for 5 minutes, resuspended in mannitol experimental buffer (MEB; 10 mM HEPES (pH 7.5), 150 mM mannitol, 50 mM KCl, 0.02 mM EGTA, 0.02 mM EDTA, 0.1% BSA, and 5 mM succinate), and added to wells of prepared 96-well plates containing the indicated peptide conditions and 0.001% digitonin. The cells were then incubated for 60 min at 28 °C, followed by fixation for 15 minutes in 8% PFA. Fixation was neutralized using N2 buffer (containing 1.7 M tris base and 1.25 M glycine, pH 9.1), and the cells were subsequently stained overnight with DAPI and an Alexa Fluor 647-conjugated anti-cytochrome c antibody (Biolegend, 612310) at 4 °C. Finally, the stained cells were analyzed using an Attune NxT flow cytometer.
Instrument	Flow cytometry was performed using a NovoCyte Quanteon analyzer (Agilent Technologies, Santa Clara, CA, USA), a Beckman Coulter Cytoflex S and an Attune NxT flow cytometer.
Software	Data were analyzed using FlowJo™ v10.10 Software (BD Life Sciences) and Beckman Coulter Cytoflex S and Attune NxT flow cytometer system
Cell population abundance	10000 cells were analyzed for fluorescent intensity in the defined gate
Gating strategy	*Describe the gating strategy used for all relevant experiments, specifying the preliminary FSC/SSC gates of the starting cell population, indicating where boundaries between "positive" and "negative" staining cell populations are defined.*

- [x] Tick this box to confirm that a figure exemplifying the gating strategy is provided in the Supplementary Information.
